# Human-centred design in global health: A scoping review of applications and contexts

**DOI:** 10.1371/journal.pone.0186744

**Published:** 2017-11-01

**Authors:** Alessandra N. Bazzano, Jane Martin, Elaine Hicks, Maille Faughnan, Laura Murphy

**Affiliations:** 1 Taylor Canter for Social Innovation and Design Thinking, Tulane University, New Orleans, United States of America; 2 Creative Social Change, London, United Kingdom; 3 Tulane University School of Public Health and Tropical Medicine, New Orleans, United States of America; Universita degli Studi di Firenze, ITALY

## Abstract

Health and wellbeing are determined by a number of complex, interrelated factors. The application of design thinking to questions around health may prove valuable and complement existing approaches. A number of public health projects utilizing human centered design (HCD), or design thinking, have recently emerged, but no synthesis of the literature around these exists. The results of a scoping review of current research on human centered design for health outcomes are presented. The review aimed to understand why and how HCD can be valuable in the contexts of health related research. Results identified pertinent literature as well as gaps in information on the use of HCD for public health research, design, implementation and evaluation. A variety of contexts were identified in which design has been used for health. Global health and design thinking have different underlying conceptual models and terminology, creating some inherent tensions, which could be overcome through clear communication and documentation in collaborative projects. The review concludes with lessons learned from the review on how future projects can better integrate design thinking with global health research.

## Introduction

The present scoping review aimed to provide timely detail on how human centred design is being applied to health outcomes research, and what the results are thus far, in order to inform future efforts at applying design thinking to global public health. Improving global health is one of the most complex and urgent social challenges of our time, and is inherently linked with economic development, improved governance, sustainable environmental strategies, and improving financial wellbeing and quality of life for all people.

In 2003, writing in the Stanford Social Innovation Review, researchers defined social innovation as: ‘the process of inventing, securing support for, and implementing novel solutions to social needs and problems’ [1]. In a subsequent article 5 years later, Phills and colleagues revised the definition to ‘a novel solution to a *social* problem that is more effective, efficient, sustainable, or just than existing solutions and for which the value created accrues primarily to society as a whole rather than private individuals’ [2]. Many conceptual frameworks exist to foster social innovation and tackle the inherently complex social problems that it addresses. The focus of the current review is on one of those frameworks, alternately referred to in this review as design thinking or human centered design. The term “human centered design” has evolved over time and originated in the fields of ergonomics, computer science and artificial intelligence, and can be seen in the international standard ISO 9241–210 which describes ‘approach to systems design and development that aims to make interactive systems more usable by focusing on the use of the system and applying human factors/ergonomics and usability knowledge and techniques’ [3]. This early engineering-related definition presumed an intended and predetermined use for each item/service as well as a static “user” envisioned by the designer/engineer [4]. However, Giacomin notes that this early concept of human centered design as the science of human use and interaction with predefined objects or services has over the years evolved into its more recent and complete manifestation as a design paradigm based on human behaviors and meanings, which he states, ‘is based on the use of techniques which communicate, interact, empathize and stimulate the people involved, obtaining an understanding of their needs, desires and experiences which often transcends that which the people themselves actually realized’ [4].

Design *thinking* uses a “designer-ly” mindset—constructive, experiential and rooted in the needs and context of end-users of a product or service—to develop novel solutions. [5] It can be seen as collectively revolving around several core concepts including empathy with users, a discipline of prototyping to gain insights, and tolerance for both ambiguity and failure [6].

User-centred design (UCD), initially termed “user centered systems design” by Norman and Draper [7] grew out of work completed at the University of California San Diego at the intersection of psychology and artificial intelligence, and is deeply rooted in human computer interaction. Since this early conception, the field has seen the incorporation of more participatory research methods, and the evolution has occurred as the design approach has been adopted more widely and across other fields, for example deploying the concepts of participatory and co-design, and the subsequent literature and peer-reviewed publications related to those [8–11].

A large number of tools exist for undertaking UCD research, and these may overlap with tools used in HCD/DT—the field has grown to encompass application beyond only human computer interaction and has been adopted more widely to indicate a design process that is focused on the needs and preferences of the end-user of a service or product. It is described as both a philosophy and a set of methods wherein which end-users influence and are involved in design [12]. The review includes UCD as category that may fall under design thinking, and thus we reviewed studies that described UCD where they also applied broader understanding of design thinking or HCD principles.

Applying social innovation to global public health, which is both a social need and a social problem, therefore fits well with the model described by Phills and colleagues. Human centered design or design thinking, with a focus on empathy, context, ideation and iteration, in turn appears well-suited to addressing issues of population health, and over the last decade there have been increasing examples of the use of design thinking for global health. Several recognized experts in the fields of design and health have advocated for increased crossover between the fields and described the potential role of social innovation to improve population level health outcomes [5, 13–16].

This has been highlighted in the design, business and innovation fields, where leading design firms such as IDEO, Frog and Dalberg/DIG are active in and have promoted further incorporating HCD into solving complex social problems related to health, development, and wellbeing [5, 17–20]. There are examples of human centred design for healthcare management [21, 22] and some major healthcare organizations in the United States have their own internal innovation units that incorporate design thinking into everyday practice settings [23, 24]. Design thinking has also been incorporated into education for health care workers and public health practitioners including public health students [25], medical professionals [26], and for evaluation of interprofessional health education [27].

Despite the enthusiasm for social innovation and related concepts, there is less empirical evidence of the impact of these activities on health outcomes themselves. A recent systematic review aimed to assess evidence from published empirical research on the impact of social enterprise activity on health outcomes. Five studies met the inclusion criteria. The authors reported that included studies provide limited evidence that social enterprise activity can impact positively on mental health, self-reliance/esteem and health behaviors, reduce stigmatization and build social capital, all of which can contribute to overall health and well-being. They noted a clear need for research to better understand evidence causal mechanisms upon a range of intermediate and long-term public health outcomes [28].

In line with the aim of the research, the format of a scoping review was selected as the most appropriate methodology [29]. The purpose of a scoping review is to survey previous research activity, find gaps in the research, and where appropriate, to decide the value of conducting a full systematic review [30]. Generally, scoping studies may establish the key themes that comprise a research area, along with the main locations and categories of information in areas that have not been examined extensively. In contrast with systematic reviews, scoping studies do not seek to identify gaps in research due to issues of quality, but rather serve a preliminary function in a more detailed hierarchy of review methods [29, 30]. The review was adapted from both Peters’ [31] and Arksey and O’Malley’s guidelines for scoping reviews [30] though these were interpreted within an interdisciplinary framework to allow for flexibility. The stages described by the guidelines may be iterative and are roughly comprised of the following: identifying the research question to be addressed; identifying works that are of relevance to the research question; screening and selecting works to be included in the review; charting the information and data within the included studies; and collating, summarizing, and reporting results of the review.

The interdisciplinary nature of the review necessitated a broad approach which focused on current and germane research to the topic at hand rather than an evidence-based, systematic review approach more typical in public health and biomedicine, as much of the work of design thinking is applied work carried out at community level by transdisciplinary teams. Per the guidelines of Arksey and O’Malley, we attempted to consider all aspects of the research area prior to formulation of the research question, to allow sufficient breadth. For example, we have included a much broader approach to searching both published and non-published literature at the earliest stages.

A very useful aspect of scoping reviews can be the ability to bridge literature in diverse disciplines or those disciplines with emerging evidence, as scoping reviews are not solely focused on the effectiveness (and hence evidence on) a particular approach to health or intervention. Per Anderson and colleagues [32], scoping reviews may be useful in mapping a body of literature with relevance to time, context/location, source (including grey literature), and academic/disciplinary origin.

Following our initial scan of the literature and discussions, research questions were formulated by the research team which consisted of public health researchers, information technologists, international development practitioners and scholars. The following research questions were identified:

What research is currently available in the published and grey literature on design thinking/HCD for health outcomes? What research is available within the following content areas on DT/HCD: public health, biomedicine, social innovation, development, and business? Why and how can HCD be valuable in the contexts of health outcomes and related research, for whom, and in what circumstances?

## Methods

The authors initially consulted with two librarians, one a medical and health specialist, another specialized in social science and business. Pilot searches were conducted at length in several domains and within both databases and grey literature. A public health research librarian (EH) developed a final list of the most appropriate search terms and strategies, which were applied to a broad selection of databases intended to reflect the nature of the scoping review and the transdisciplinary requirements of social innovation literature. The initial search was limited to documents written in English and published from 2006 to 2016. This time frame selected was 2006–2016, as there has been an increase in interest in the application of human centered design and design thinking to health outcomes over the past decade. The literature search took place from June-August 2016.

For the first stage of the literature search, peer-reviewed literature was sought to contribute to answering the question, “What research is currently available in the published public health, biomedicine, social innovation, development, and business literature on design thinking/human centered design for health outcomes (programs, interventions or projects for the creation or improvement of health)?” One exemplar article was available, a case study of the use of HCD for public health innovation: Vechakul, J., Shrimali, B. P., & Sandhu, J. S. (2015). Human-centered design as an approach for place-based innovation in public health: A case study from Oakland, California. *Maternal and Child Health Journal*, *19*(12), 2552–2559. doi:10.1007/s10995-015-1787-x [doi]. Systematic reviews were sought using PubMed Clinical Queries and the Cochrane Database of Systematic Reviews.

Three health bibliographic databases were selected to locate health science literature: PubMed, EMBASE, and CINAHL. A keyword search was developed for the terms "Design Thinking" OR "Human Centered Design" OR User Centered Design" AND outcome. PubMed *PubReminer* was used for citation pearl-growing and ultimately 697 de-duplicated results were located. A slightly different keyword search, which included the term ‘health outcome’ was developed for each of 15 non-health bibliographic databases, including three citation indexes, to locate literature about HCD/DT and health outcomes from the disciplines of business, economics, sociology, social work, and anthropology. Databases included SCI-EXPANDED, SSCI, and Arts & Humanities (Web of Science), ABI/INFORM Global, ASSIA, Dissertations and Theses, PAIS, Sociological Abstracts, Social Services Abstracts, Social Science (ProQuest) and Anthropology Plus, Business Source Complete, EconLit, SocINEX, Social Work Abstracts (EBSCOHost).

Searches in Web of Science SCI-EXPANDED, SSCI, and Arts & Humanities, ProQuest ABI/INFORM Global, Dissertations and Theses, Sociological Abstracts, Social Services Abstracts, Social Science, and EBSCOHost SocINDEX ultimately returned 175 de-duplicated results. Searches in ProQuest ASSIA, PAIS, AnthropologyPlus, BusinessSource Complete, EconLit, and Social Work Abstracts returned no results. Please see **Appendix 1** for the full details of the search strategy in each database.

### Grey literature

In developing the grey literature search plan, a variety of searching strategies were considered and ultimately utilized: 1. customized Google search engine, 2. targeted websites, 3. consultation with experts, 4. hand search by the research team. The implementation of complementary strategies allowed the researchers to minimize the risk of leaving out pertinent documents and important sources of applied work. Where abstracts were not available in grey literature documents, authors reviewed executive summaries or table of contents for screening, followed by full-text screening.

Two reviewers independently screened titles and abstracts for eligibility.

### Criteria and selection

Documents were included which presented information about the application of HCD or design thinking to health outcomes, defined as the creation or improvement of health among humans. Studies published or made available in the English language within the last 10 years were included.

Studies that met inclusion criteria used widely accepted research methods and had well described study methodology, including but not limited to the following: case studies, evaluations, ethnography, intervention research, randomized trials. Included studies provided a clear description of recognized data analysis methods, including applied methods, and described the results of the project, including any immediate or long-term impact the project may have had.

**Fig 1:**
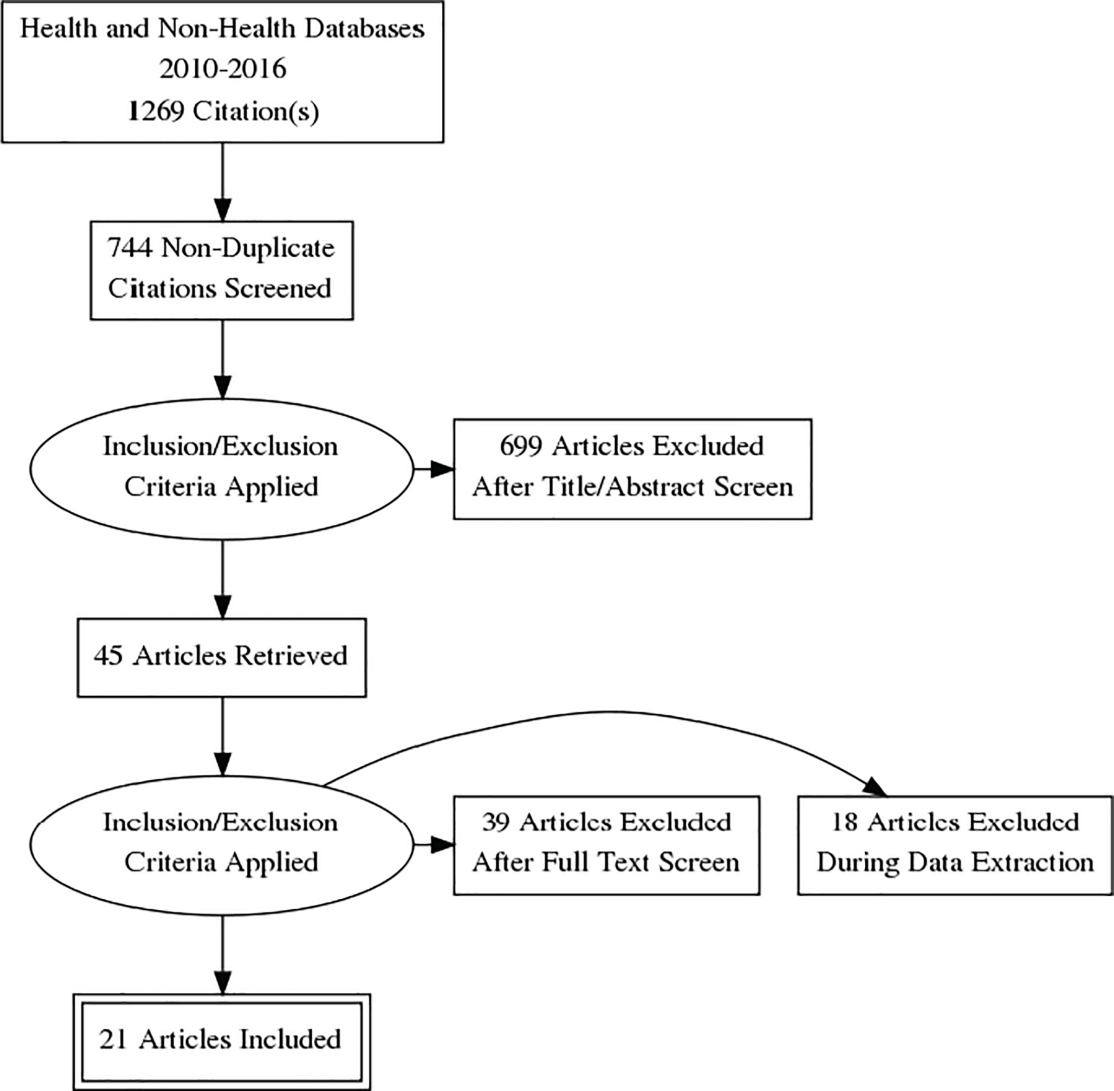
Data flow during review process.

The researchers excluded studies where it would be difficult to chart or extract data for the purposes of a review, e.g. studies where the design thinking/HCD/UCD approach could not be clearly identified, such as summaries or aggregate data on design thinking or social innovation, and those studies which did not directly pertain to human health outcomes. Commentaries, protocols and abstracts from conference proceedings (wherein sufficient information on the application of HCD/DT for health could not be extracted) were also excluded. A full illustration of the selection process is presented in **Fig 1**below. During the process of applying the inclusion and exclusion criteria, it was noted that a significant number of documents described the use of UCD only for improving on an already developed health technology such as a clinical decision tool, software application, computer interface, or similar projects. The determination was made to exclude studies that used UCD only as a means to improve human computer interaction related to a health technology, as opposed to the development of a new health technology or services.

### Charting the data

The research team used a multi-stage, iterative process to chart the data. Four reviewers (LM, JM, MF, AB) utilized a standardized form tailored to the needs of the review to chart data from the included documents. Reviewers began by extracting common descriptive information from individual articles based on specified categories: author(s), publication date (year), title of the document, source (journal, database), aims of the study, study design or literature descriptors (research study, applied use, case study, evaluation), location of the study, study participants, sample size if applicable, theoretical approach, design approach used, data collected (outcomes, measures reported), key findings reported, and reviewer comments. In some cases, documents thought to be appropriate for inclusion were removed at the stage of full review for data charting. Grey literature was also reviewed but in many cases documents were unsuitable for charting, due to missing elements (e.g. methods used, participants, outcomes). A second round of charting was done with the documents selected for inclusion using a smaller standardized form, containing fewer items. This is presented in the results section.

## Results

Ultimately, 21 documents were identified from database and grey literature searches and were charted for review. These are presented below (see **Table 1**).

**Table 1:** Results of scoping review.

Author/ Source	Participants/ Setting	Use of HCD/DT	Health Outcomes	Methods/ Activities	Results Reported
**Almon, N.** [33], *Thesis for Master of Science*	Chronically ill emerging adults (18–26 yrs old) in the US	Design thinking or human-centered design was utilized as the vehicle to discover unmet 'emerging adult' and adolescent health clinician needs.	Disease self-management skills and healthy lifestyle behaviors	Ethnography (observations, interviews), survey, literature review	Five design recommendations were suggested to ensure that the outpatient clinic supported both clinician and 'emerging adults' needs.
**Amiri, M.,** ***et al***. [34], *Work (Journal)*	School-going children 7–9 yrs old in Iran	User Centred Design was used to design a backpack that decreased load on shoulders, neck, waist.	Musculoskeletal disorders and pain, postural dysfunction	Ethnography (observation, interviews), focus groups, hidden filming, prototyping, brainstorming	UCD approach solved ergonomics approach for health considerations and addressed user preferences for aesthetics and appearance.
**Catalani, C.** ***et al***., [35] *PLOS One (Journal)*	People impacted by TB and HIV, those who care for them, and administrators or staff of a care organization in western Kenya	HCD was explicitly used to (1) understand the situation through the collection and analysis data; (2) develop a new clinical decision support system, (3) implement and evaluate the system across 24 clinics.	TB and HIV	Ethnography (site observations, key informant interviews), lab simulation, and in-context usability testing	HCD facilitated the process of digital innovation in a complex and resource-constrained context; improved understanding of the needs and assets of providers; created a TB clinical decision support system to improve intensive case-finding and IPT initiation among patients living with HIV, and implemented the system.
**Cheney, C.** [36], *Electronic Article*	Unmarried adolescent females in Tanzania and PSI staff and leadership who serve these clients	HCD processes used to identify new channels to provide contraceptive services.	Contraception for unmarried adolescent females	Design immersion, brainstorming, interviews, observation, prototyping with end users	Developed new channels for providing contraceptive information and services, plan to scale up HCD methods organization-wide.
**DeVoe, J.** ***et al*.** [37] *The Journal of Ambulatory Care Management*	Children (aged under 18 yrs.) their families, medical informaticists, federally qualified health center (FQHC) staff, and researchers in the US	User-centred design used to build and test customized information technology tools to help FQHCs reach uninsured children and those at risk for losing coverage.	Children without health insurance and those at risk for losing health insurance coverage	Collected qualitative data (e.g., observation of clinic, family and staff interviews), research team spent approximately 95 hours directly observing 4 sites and conducted one-on-one interviews with 19 families and 31 FQHC staff members	Developed HIT tools to create “pop-up” alerts to remind health center staff to talk with patients about insurance status and upcoming deadlines for reapplication, and automate registries of children who are uninsured or nearing insurance expiration dates.
**Fahnrich, C.** ***et al***. [38] *Eurosurveillance (Journal)*	Stakeholders in Nigeria involved in Ebola outbreak control, epidemiologists and designers	Design thinking was used to analyse experiences of Nigerian field workers and the Ebola Emergency Operations Center.	Ebola control	Design thinking workshops, development of personas, artefacts and process models related to ebola outbreak operations.	Developed software—the Surveillance and Outbreak Response Management System (SORMAS to ensure availability of validated real-time surveillance data and to manage the verification of cases as well as tracing and monitoring of their contacts as it is typically needed during an EVD and other disease outbreaks.
**Knoblock-Hahn and LeRouge** [39] *Journal of the Academy of Nutrition and Dietetics*	Parents of overweight and obese female adolescents (aged under 18 yrs.) in the US	UCD application in a qualitative study that sought to determine parental views on how technology can support previously learned behaviors that require ongoing management and support beyond formal lifestyle interventions.	Overweight and obesity in adolescents	Collected qualitative data (14 interviews) with parents of overweight and obese female adolescents	Applications that teach adolescents how to cook were described as ideal for shared use between parents and adolescents because they are supportive of the role of the reciprocal causation for eating behaviors in the home.
**Koehly, L** ***et al***. [40] *BMC Public Health (Journal)*	Mothers (≥18 yrs.) with young children in the US	UCD steps taken to develop and evaluate a workbook used as an educational tool outlining family health history based risk of heart disease, type 2 diabetes, breast cancer, and colorectal cancer.	Reduced risks of heart disease, diabetes, breast cancer and colorectal cancer that cluster in families	Collected qualitative data (interviews and focus groups) with mothers of young children with assessment and follow-up	*The design of the workbook was refined in response to participant feedback and subsequently re-evaluated*. *Results*: understanding of the workbook components improved for all sections, 100 % of users were able to use it to assess their disease risk and >60 % to assess family members’ disease risk. Participants had better confidence to increase fruit, vegetable and fiber intake improved significantly, as well.
**McCreary, L.** [41] *Harvard Business Review (Journal)*	Kaiser Permanente patients, staff, and administration in the US	Design Thinking is used by the Innovation Consultancy, a small team within the health care provider Kaiser Permanente, practicing an expansive, service-focused version of innovation that is both rapid and economical.	Care and management of Kaiser Permanente’s 8.6 million patients	Innovation lab uses ethnography, observation, deep dives, co-design.	KP Medrite project resulted in reduced costs associated with medication errors, greater employee satisfaction and patient peace of mind. Nurses Knowledge Exchange resulted in handover at the patient’s bedside rather than at the nurses’ station and introduced new software. Innovation Learning Network has been developed to share innovation beyond Kaiser Permanente with other healthcare organizations.
**Moody, L.** (12] *Journal of Medical Engineering and Technology*	Devices for Dignity Healthcare Technology Co-operative, users of health services, and stakeholders in the UK	UCD process brings together Industry, Academia and the NHS to design and develop innovative technology solutions to support people with long-term conditions.	*Management of long-term health conditions*: assistive and rehabilitation technology; renal technologies, urinary continence management and paediatrics	Empathic approach with users who have long-term health conditions. Interviews and focus groups, prototyping, user testing and iteration, co-design and surveys	*Examples of completed projects*: leg-worn urine drainage bag; innovative shower chair to meet the needs of the active, independent, self-purchasing wheelchair user; prototype urinary catheter; prototype urinary catheter incorporating complex equipment needs such as ventilators and oxygen cylinders.
**Morrison *et al*.** [42] *BMC Medical Informatics (Journal)*	Adults (18 yrs. and older) with asthma and practice nurses involved in asthma management in Scotland	Multifaceted processing incorporating a UCD process for development of online intervention for asthma self-management.	Asthma self-management	Focus groups and think aloud study, prototyping	Online internet intervention for behavior change and self-management of asthma developed and will undergo evaluation in a randomized controlled trial.
**Mummah, S.A**. [43] *The International Journal of Behavioral Nutrition and Physical Activity*	Collaborative academia-industry partnership of researchers, product designers, engineers, and dietitians in the US	IDEAS framework guided the process and was used for its integration of behavioral theory, User-Centered Design and Design Thinking, and evaluation.	Dietary self-monitoring for improved nutrition	Qualitative interviews, ideation, prototyping, user testing	Pilot RCT findings suggested the initial efficacy, acceptance, and feasibility of the intervention. The final version of Vegethon enabled easy self-monitoring of vegetable consumption and included a range of features designed to engage participants.
**Papi, E. *et al*.** [44] *BMJ Open (Journal)*	Adults with osteoarthritis (age range 45–65 years) in England	UCD approach adopted to develop a rehabilitation tool for patients with osteoarthritis.	Osteoarthritis rehabilitation	Focus groups, prototyping	Identified determinants of user acceptance of a wearable technology and reported patient preferences and information derived from the research.
**Ramos, A.K. *et al*.** [45] *Progress in Community Health Partnerships (Journal)*	Spanish-speaking Latina women in Nebraska, US	Design thinking was used to create a health education specifically designed for monolingual Spanish-speaking immigrant Latinas in Nebraska.	Health education for immigrant Latina women	Co-creation, co-design, dialogue, prototyping, brainstorming	Increase in women’s health knowledge based on data from pre-test and post-test surveys.
**Robinson, L. *et al*.** [46] *International Psychogeriatrics (Journal)*	People with dementia and their carers in the UK	UCD/Participatory design used to create acceptable and effective prototype technologies to facilitate independence for people with dementia	Dementia care	Focus groups, workshops, prototyping	Prototypes for two devices (armband and electronic notepad) were developed. The study showed that involving people with dementia in the process of participatory design is feasible and could lead to devices which are more acceptable and relevant to their needs.
**Sanematsu, H**. [47] *Journal of Adolescent Health*	Young adolescents in Indiana, US	Designers trained in UCD developed relevant and informative communication materials to raise awareness about adolescent health issues.	Adolescent health issues	Focus groups	Prototyping and final development of public service announcements (PSAs] and a health survival booklet.
**Sax *et al***.[48] *Journal of Hospital Infection*	Health care workers in Switzerland	UCD approach incorporating strategies of human factors engineering, cognitive behaviour science and elements of social marketing, followed by an iterative prototype test phase within the target population.	Hand hygiene / disease transmission	Prototyping	Presented the results of the work (“My 5 moments for hand hygiene concept”] but no specific details reported on the process used.
**Seeber, L.** [49] *Current Drug Safety (Journal)*	Students of the School of Design Thinking in Potsdam, Germany and staff of The Vienna Vaccine Safety Initiative	*Design thinking was used to address the question*: “How might we enable physicians to encourage parents and children to prevent infectious diseases?” over the course of a 12 week project.	Vaccine safety	Field research, including interviews with international experts, parents, pediatricians, and children, generation of representative Personas, Prototyping	Developed VAccApp™, a digital vaccination record helping parents keep track of recommended immunizations for their children while integrating vaccine recommendations and reminders for booster immunizations into every-day life. The app was in beta testing at the time of the publication.
**van Hoof, J**. [50] *Journal of Aging Research*	Professional stakeholders involved in care of bed-ridden nursing home residents in the Netherlands	Participatory action research and UCD with stakeholders (not residents of nursing homes themselves) to provide design guidance on improving the care environment for bed-ridden nursing home residents.	Mobility and activities of daily living of nursing home residents confined to bed	*Work groups carried out*: scenario-writing, storyboarding, performance ethnography, and collaborative design and prototyping.	*Reported design solutions and suggestions developed*, *including*: the supply of technological items and architectural features; concepts and products that are available on the marketplace; and those not yet available that relate to improvements in resident autonomy and environment.
**Vechakul, J.** [51] *Maternal Child Health Journal*	14 community/public health professionals in Oakland, US	12-week pilot in which professionals from nine organizations used the HCD process to develop concepts for stimulating a vibrant local economy in the Oakland Best Babies Zone.	Infant mortality and community level health.	Design sprint, semi-structured interviews, prototyping	Informed the design of a Community Market (hosted a total of 20 vendors, generated US $3,212.60 in profit for vendors, and attracted 585 attendees) and led to creation of the East Oakland Innovators program.
**Whinnery, J.** [52] *Global Health Science and Practice*	Community members in Kisumu, Kenya (household members, teachers, health care workers)	Interactive and iterative design used to develop convenient and economical hand washing system.	Water and sanitation, hand washing	Focus groups, interviews, prototyping	In focus group discussions, approximately 80% of participants stated they would purchase a “Povu Poa” product, suggesting the aspirational value of the product. Final development of the model was reported to be taking place in the future (at the time of publication).

**Fig 2:**
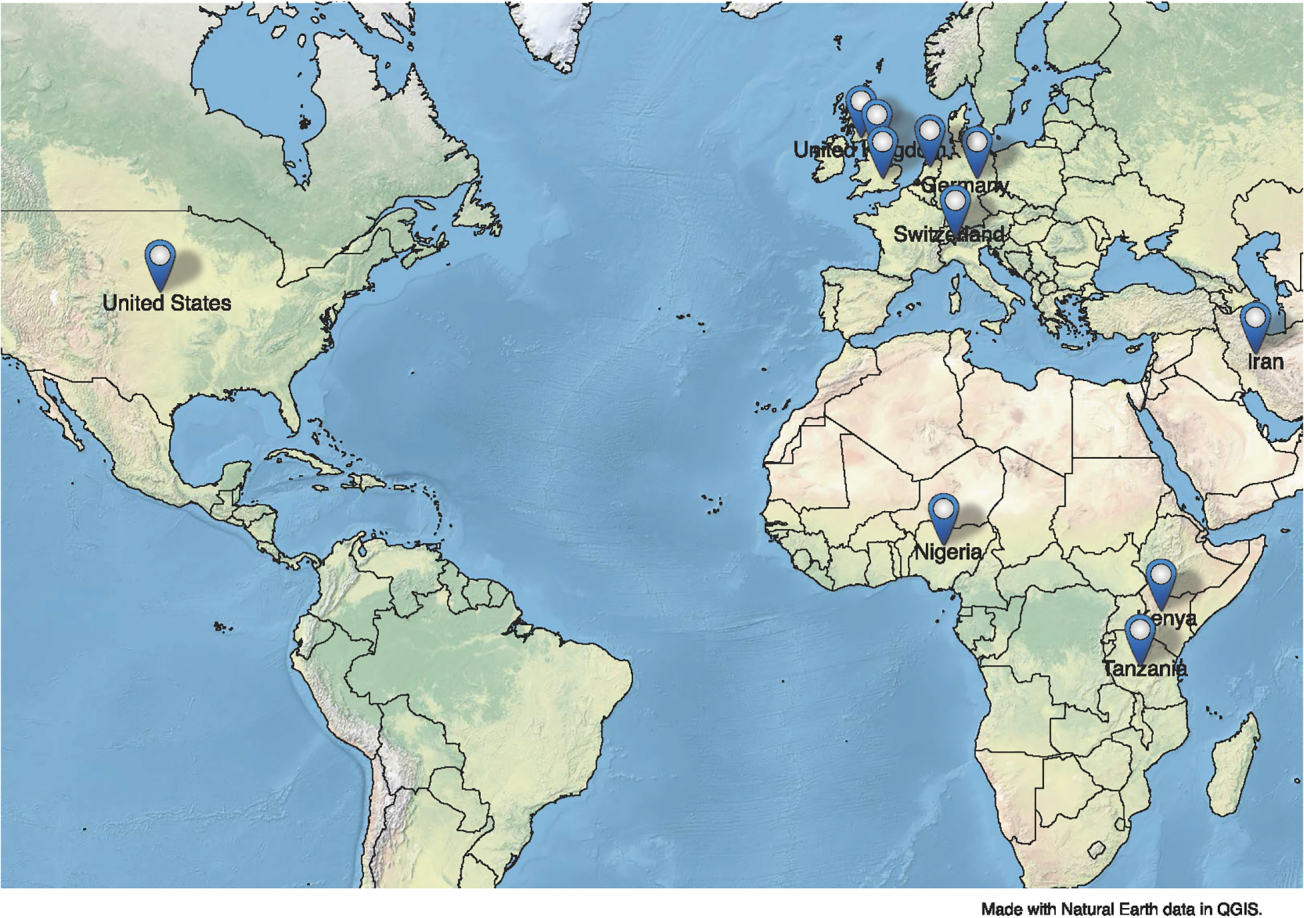
Locations of studies identified.

*Source*: *Public Domain Data (Natural Earth) and QGIS (open source GIS)* http://www.naturalearthdata.com/about/terms-of-use/

There was a wide distribution in geographic regions from which studies originated. Nine of the studies included in the review came from the United States, 4 from the United Kingdom (England, Scotland, or unspecified), 3 from Europe (Switzerland, Germany, the Netherlands), and 4 from Africa (Nigeria, Kenya, Tanzania). **Fig 2**illustrates the geographic distribution of the various research sites (where possible).

The review explored the various contexts where human centered design may be used to address health issues. These can be categorized as having a direct or indirect impact on health outcomes. Those with a direct impact included scenarios where HCD was used for designing health programs, products or health services directly utilized by a person for the creation or improvement of health. Those categorized as having an indirect impact involved using HCD/DT to improve management, organizational issues, the environment for health, or technology applications not used directly by a person for the maintenance or improvement of their own health. For example, improving identification of children who don’t have health insurance.

Of the contexts in which human centered design or design thinking was applied to human health outcomes, the vast majority related to technology and specifically health-related technology such as development of software applications, clinical decision tools, websites, and other technology platforms designed to allow for improvement of health and specifically behavior that impacts on health, whether an individual’s own health-related behavior or the behavior of someone caring for an individual’s health (such as a parent, nurse or doctor).

Documents selected for review fell under four main categories related to health: disease management related to serious or chronic illness, health systems and care management, infectious disease prevention or care, and primary prevention and health behavior/education, **Table 2**illustrates the categories and the specific applications of HCD within each category.

**Table 2:** Health applications of human centred design.

Health context	Example
**Disease management, serious and chronic health conditions**	Disease self-management for chronically ill young adults
	Reducing familial risk of heart disease, T2D, cancer
	Management of renal disease, assistive rehabilitation, urinary continence
	Asthma self-management
	Osteoarthritis rehabilitation
	Dementia care
	Mobility and ADLs of nursing home residents
**Health systems and care management**	Children without health insurance, Kaiser Permanente managed care
**Infectious disease prevention/care**	TB and HIV patients and carers
	Control and prevention of Ebola transmission
	Hand hygiene of health care workers
	Water Sanitation Hand Hygiene
	Vaccine Safety
**Primary prevention and health behavior/education**	Dietary self-monitoring
	Obesity/overweight in adolescents
	Health education for Spanish speaking immigrant women
	Infant mortality (Best Babies Zone)
	Preventing musculoskeletal disorders and pain in children
	Adolescent health issues
	Contraception

There were very few studies that described a full project cycle, and even fewer that presented the evaluation of health projects that used HCD. One notable exception was Koehly et al. who presented the results of a UCD designed-workbook for reduction of disease risk among families [40]. Catalani et. al also described an in-progress clinical trial to assess the impact of their TB clinical decision support system, for future reporting (see Table 1).

Conversely, many studies did not describe the socio-institutional dynamics of the HCD. Many studies briefly noted design team composition, capacity or the institutions responsible for design (see Table 1. DeVoe, Fahnrich, Seeber, van Hoof, Sanematsu, Vechakul, McCreary, Cheney), but some did not mention or analyse the design team’s background, involvement, or relationship to context or users. This omission was particularly evident in studies following more typical public health standards, of researcher-led design processes. For example, though Koehly et. al documented product development and results, there was no discussion of *who* was involved in development (see Table 1.).

More than half of the studies featured design teams with non-design professionals on them. However, there were exceptions: though all the African-based projects were led by majority Western teams, Catalani et. al mentioned the design team’s status as outsiders (see Table 1. Catalani). The study by Vechakul et. al. also focused overtly on the design process and design-thinkers-in-training as research subjects [51].

A wide range of engagement processes were evident in the studies reviewed, but less attention to differences was also noted. Most studies described interaction with users, but some conducted no immersion or testing with real people. Some described co-design or participatory processes (see Table 1. van Hoof, McCreary), but this label was applied differently across studies. For example, for Catalani et. al, HCD was described as a participatory process because it involved immersive research with real users (see Table 1.); this is reminiscent of participatory rapid appraisal methods [53]. For van Hoof et. al., the process did not include end-users (only stakeholders) in the co-design sessions (see Table 1.).

## Discussion

The scoping review identified a number of documents describing the use of HCD for health, but also revealed pervasive gaps in both published and unpublished literature, especially related to replicable methods, description of methodologies used, evaluation of effectiveness or impact of HCD projects on health, and lifespan of design thinking projects along with potential for scale up of these initiatives for health.

Based on the large number of references to design and innovation in health, found through the searching the grey literature, it is evident that various initiatives are underway across many different types of organizations (international development agencies, public health agencies, hospitals and health systems) to use HCD or DT for the creation or improvement of human health. However, very few of these initiatives were clearly described and documented in a way that would allow for reporting in a scoping review. Most did not provide detailed information on methods or results, and many did not include timeframe or other details, making it difficult to report on or quantify the extent of use of these types of tools.

In almost all documents reviewed, design thinking was used to adapt or improve an existing approach to creating or improving health, rather than developing a new approach altogether. This is in accordance with a recent review of innovation more generally in the field of global maternal and child health [14].

### Lessons learnt in reviewing the literature

In the course of conducting the review, several insights related to scoping the literature on HCD and health were noted and are presented here. These may inform future reviews in this area and may allow for research to be adapted to best locate literature related to these topics.

HCD and design thinking concepts, as applied to health, are sometimes difficult to define, and therefore to systematically review. Definitions of human centred design and design thinking vary from organization to organization and are not standardized (e.g. Stanford d. school, the design consultancies IDEO and Frog all have different definitions), so reporting and disseminating information about projects that use these methods can be difficult. There are many practices which take these names, and many activities which fall under an authors’ definition of HCD, but are not named as such, and thus are difficult to discern and categorize. No widely accepted definition exists within the broader design community on the essential characteristics that make DT/HCD different from other design or participatory practices (e.g. service design or Community Based Participatory Research), but rather varying definitions are used.

Terms related to HCD and health are hard to search for in diverse bodies of literature (e.g. business, sociology, public health, design), and experience in searching on related terms is an important factor in successful retrieval of pertinent literature. Different fields use similar (and sometimes the same) terms, and use of terms seems to be somewhat fluid. User-centred design in some cases signifies the same as human-centred design, and in some cases has a focus on human computer interaction. This insight holds particularly true for database searching. For example, the major biomedical database PubMed, does not have a specialized searching (MeSH) term for design thinking or human centred design. A syntax that catches all terms may work in one database but not in others, and information technology may or may not be able to control for these differences. ‘Design’ and ‘health’ are words that have many meanings and are used in different ways across the various literatures that we searched, thus they turned up a large number of irrelevant results (e.g. ‘the health of the business’, ‘design of organizations’). HCD projects and researchers can provide further clarity to the discussion by documenting their methodological choices.

An inherent tension exists between the way research is undertaken in design versus in the health sector, particularly related to population-level health research and this impacts reporting on of research. Many of the central tenets of design thinking research, like iteration, tolerance for ambiguity, pivots, and rapid prototyping, are inherently at odds with some prevailing processes in health and biomedicine, particularly public health, where hypothesis-driven research is the norm and where the evidence base (typically the peer-reviewed literature) is used to generate concepts for study.

Several authors of studies reviewed (see Table 1. Catalani and Cheney) noted concerns about HCD’s methodological rigor as compared to health research standards. For example, obtaining ethics approval to work with human subjects (even to undertake qualitative research) requires detailed information on aims, objectives, projected risks and benefits, as well as a strong evidence base and rationale for the proposed research. The process of compiling this information and validating it through peer-reviewed literature is quite different from the iterative cycles designers typically utilize when working with end users or beneficiaries. Similarly, health and biomedical research conducted through public or non-profit funding usually requires a hypothesis driven study design, based on existing theory, with planning for rigorous evaluation of the results. A funding agency such as the US National Institutes of Health or the UK Medical Research Council may prefer to fund research with clear objectives over exploratory research because hypothesis testing is considered less risky and easier to assess in terms of rigor. While some HCD projects incorporate similar processes, care needs to be taken to explicitly describe these, where they would be expected as basic tenets in health research.

Processes used to measure and evaluate the input, output, process and impact of a project are very different in the two fields. The creation of a new tool (e.g. a product or service) to improve public health may be considered an outcome from a design perspective, but would not be considered a health outcome from a public health perspective, where the key outcome of interest would be the impact of the tool on public health. Similarly, designers may prioritize usability or acceptability, and may not be involved at later stages where impact is evaluated or projects are scaled up. Moody noted the particular challenges to undertaking user-centred design in healthcare, which must be balanced against the benefits [12]. In particular, this highlighted the need for expertise from diverse fields, along with the input of end users, a collaboration that may not be simple or straightforward, but which will ensure the best solutions. To that may be added the involvement of designers from start to finish in designing and then eventually evaluating the health impact of an innovation.

Much of these differences may be related to the variance of use of design thinking in practice related to health, and use of design thinking (or social innovation methods more broadly) in research related to health. Catalani and collegues (see Table 1.) noted that HCD is not a research methodology per se. HCD is closer to what Manzini calls ‘research through design’, a more subjective process using research methods to generate concepts or ideas that is often practitioner-led [54]. This is different from ‘research on or for design’ which investigates the process or nature of design to generate, which is different still from scientific investigation aiming to understand natural or social phenomena [55]. Some of the studies reviewed have combined these different modes of research, combining user-centered research, descriptions and insights from the design process, knowledge from fields like ergonomics, neuroscience and economics, with public health evidence for the creation of new concepts (see Table 1. van Hoof, Sax, Amiri, Catalani). This may add to the richness of HCD processes, but increase the confusion in interpretation across disciplines.

A recent Stanford Social Innovation Review article highlighted readers’ views about the role of “research” in social innovation, and the tensions between researchers and practitioners. A survey of practitioners (n = 1892), engaged mainly in applied social innovation (including HCD/DT) across various fields like health and education, sought to answer questions on how practitioners source and use research on social innovation. Many respondents preferred practitioner-oriented publications or internal research. Only a small number (16%) found academic journals as most relevant to their own work, which may be related to the difficulty in finding articles on social innovation projects in peer reviewed literature. One respondent noted, ‘academic journals are difficult to use as a teaching/discussion tool for front line staff,’ and another stated ‘there is a perception that an academic article moves the discussion to the theoretical realm and away from practical consequences.’ [56]

Describing the specific use of human centred design in a maternal child health project in Oakland, Vechakul suggested that in public health there may be ‘uneasiness with open-ended processes that have no predefined outcomes’ but went on to advocate for HCD as ‘a systematic process that helps people embrace ambiguity and generate new insights and ideas…HCD can provide a structured process to systematize innovation in public health, shorten planning timeframes, and co-create with community members and cross-sector partners’ [51]. Faculty at UC Berkeley recommend that design thinking be taught to future professionals because complex health challenges may require new problem-solving approaches beyond the biomedical evidence base [25]. Another approach suggests a somewhat intermediary role for HCD: it could be useful to increase adoption or to tailor known best-practices to a given context. This is evident in one of the reviewed study’s intention to translate scientific evidence into user-centered designs, focused on human behavior in the uptake of best practices (see Table 1. Sax).

Further, the design and implementation of HCD-based public health projects in diverse social systems and clinical settings must be considered. These will differ significantly, for example in low and middle income countries or high income countries. In settings where systems in which more conventional quality assurance healthcare services exist, these may evolve or merge with newer approaches in order to expand the ability to provide the best possible outcomes. Two early examples may be the approaches used by large health systems in the United Sates such as the Mayo Clinic [24] or Kaiser Permanente [23]. In more resource-constrained settings, it will be important to ensure that systems get implemented in order to understand the value added by HCD components in global public health projects and interventions.

Fabricant called on the design community to ‘reassess the level of value-to-inspiration in our outputs and increasingly prioritize actionable strategies that we can quickly test, refine, and integrate into operational planning.’ [13] Where this is intended to translate to health research, such actionable strategies may also need to be placed within the health literature for maximum impact. In order to serve knowledge accumulation in this area, it would be important for studies that incorporate HCD strategies and tools to clearly document the following: 1) at what stage these tools were conceptualized as part of the overall research, 2) how and by whom the decision to incorporate them was made, 3) the specific mindsets and methods employed in the project, and 4) the results or outcomes of implementing the HCD components (e.g. new ideas, results, or changes in the original plan of action).

There are few studies that describe the full life cycle of HCD/DT, from development through evaluation of the impact. Much of the literature we reviewed either provided a limited amount of information on design thinking applied to a health project, or advocated more generally for the use of HCD for health or use design thinking to develop general recommendations that may or may not be utilized. For example, Matheson called for the use of HCD for improving chronic disease prevention efforts [15], and underscored the point by describing an international consensus statement endorsing this position [16]. Similarly, Lin highlighted the potential utility of HCD for improving microbicides to prevent sexually transmitted infections [57]. These articles were commentaries, and thus were not included in the final selection. Such commentaries, while important for awareness raising, do not provide actionable strategies to achieve impact, nor a replicable model for integrating HCD directly into disease prevention efforts.

This general trend may reflect the short timeframe over which design has been applied to health and the need to measure health outcomes over long periods of time, or it may reflect a bias toward publication and promotion of only projects that have had positive results. Another possibility is that designers and researchers working in the health field see their work as compartmentalized, resulting in missed opportunities for collaborative and comprehensive approaches.

Overall, authors did not address the researcher-instrument perspective inherent in qualitative research, missing an opportunity to analyse their role in the study. If design and creativity are subjective [54], and HCD is meant to be done in interdisciplinary teams, to achieve contextually relevant designs [17], then these factors could be crucially important in shaping design process and ultimate outcomes.

### Strengths and limitations

Strengths of the scoping review methodology include that it has provided the first wide-ranging synthesis of literature on HCD and health, that it has also identified gaps in the literature and areas for future review and synthesis, and that the findings can be directly applicable to research and practice in this area. The present study also had limitations, as well as challenges particular to reviews of literature that crosses disciplinary and academic/non-academic boundaries. The inclusion of English language only documents and a ten year bound for searching could have excluded relevant documents. As the grey literature was vast, some relevant documents from it may have been excluded unintentionally. Additionally, given the broad bodies of literature searched, the large number of initial documents identified, and the relatively undefined terms required to be searched within those bodies, it proved difficult to fit the systematic nature of the scoping review framework. For example, the inclusion and exclusion criteria may have excluded articles initially screened at the abstract stage, that could have been usefully examined in their entirety. These criteria may have omitted articles that could provide more diverse examples of HCD for health. We note that most studies included came from Western contexts, which may result in a limited perspective on the application of HCD to global health in our discussion. The omission of other terms, such as service design and participatory design, which were conceptualized as approaches rather than overarching frameworks, may similarly have limited the findings. Finally, we initially intended to include all literature describing User Centered Design methods, but eventually excluded documents that described UCD methods used only for improvement of human computer interaction, a large and important body of literature, but one that seemed to relate to health indirectly.

## Conclusion

To date, this is the first scoping review disseminated in the area of HCD and health, and the results are likely to be useful for practitioners, researchers and consumers alike. The review sought to identify and describe literature related to the use of design for health, and the results presented HCD as it has been applied to health across various geographies, settings and health issues. In undertaking the review, we also identified gaps in the literature, particularly where most design studies may not adequately describe methodology, results, and impact of the application of design to health outcomes, potentially limiting the extent to which they may be critically evaluated and replicated. Future research priorities include defining and clarifying quantifiable outcomes of HCD health research and adapting common goals for the different disciplines. More rigorous evaluation of HCD as it applies to health in the future will allow for more acceptance and integration of design into health research, and ultimately the improvement of health projects through design thinking.

## Appendix 1. Detailed Database Search Information

**Basic search terms used in health databases**:

"Design Thinking" OR "Human Centered Design" OR User Centered Design" AND outcome in health databases

**PubMed Search Strategy on July 11, 2016**

1. Design Thinking"[All Fields]
2. Human-Cent* Design [All Fields]
3. “human centrifuge’[All Fields]
4. “human centrifuges” [All Fields]
5. “human centrin” [All Fields]
6. “human centrins” [All Fields]
7. “human centrioles”[All Fields]
8. “human centroblasts” [All Fields]
9. “human centromere” [All Fields]
10. ‘human centromeres” [All Fields]
11. “human centromeric” [All Fields]
12. “human centrosome” [All Fields]
13. “human centrosomes” [All Fields]
14. #2 NOT #3—#13
15. User-Cent* Design [All Fields]
16. #1 OR #14 OR #15
17. outcome* [All fields]
18. #16 AND #17

**PubMed Clinical Query Search Strategy on July 11, 2016**

1. systematic[sb]
2. "Design Thinking" [All Fields]
3. "Human Centered Design" [All Fields]
4. "Human Centred Design" [All Fields]
5. #3 OR #4
6. "User Centered Design" [All Fields]
7. "User Centred Design" [All Fields]
8. #6 OR 7
9. #1 AND #2 OR #8
10. Outcome* [All Fields]
11. #9 AND #10

**CINAHL (EBSCOHost) Search Strategy on July 11, 2016**

S1. “Design Thinking”
S2. “human Cent*Design”
S3. “User Cent* Design”
S4. S1 OR S2 OR S3
S5. “Outcomes or Effects”
S6. S4 AND S5

**EMBASE on July 11, 2016**

1. “Design-Thinking”
2. “Human Cent* design”
3. "User Centered Design”
4. #1 OR #2 OR #3
5. “'outcome assessment/exp/mj
6. #4 and #5

**Basic search in non-health databases**:

"Design Thinking" OR "Human Centered Design" OR "User Centered Design" AND health* outcome*

**Web of Science Index: SCI-EXPANDED on July 11, 2016**

1. (TS = ("Human-Cent* Design" OR "Human Centered Design" OR "Human Centred Design") “Human Cent* design”
2. (TS = ("User-Cent* Design" OR "User Centered Design" OR "User Centred Design")
3. (TS = ("Design Thinking"))
4. #3 OR #2 OR #1
5. (TS = outcome*)
6. #5 AND #4
7. (TS = health)
8. #7 AND #5
9. #8 AND #4
10. (TS = health*)
11. #10 AND #5
12. #11 AND #4

**Web of Science Index: SSCI on July 11, 2016**

1. (TS = ("Human-Cent* Design" OR "Human Centered Design" OR "Human Centred Design"))
2. (TS = ("User-Cent* Design" OR "User Centered Design" OR "User Centred Design"))
3. (TS = ("Design Thinking"))
4. #3 OR #2 OR #1
5. (TS = outcome*)
6. #5 AND #4
7. (TS = health)
8. #7 AND #5
9. #8 AND #4

**Web of Science Index: Arts & Humanities on July 11, 2016**

1. (TS = ("Human-Cent* Design" OR "Human Centered Design" OR "Human Centred Design"))
2. (TS = ("User-Cent* Design" OR "User Centered Design" OR "User Centred Design"))
3. (TS = ("Design Thinking"))
4. #3 OR #2 OR #1
5. (TS = outcome*)
6. #5 AND #4
7. (TS = health)
8. #7 AND #5
9. #8 AND #4

**ABI/INFORM Global (ProQuest)** on July 11, 2016

((all(Design PRE/1 Thinking) AND la.exact("English") AND pd(>20060711)) OR ((all(Human-Cent* Design) AND pd(>20060711)) OR (all(Human-Centered Design) AND la.exact("English") AND pd(>20060711)) OR (all(Human-Centred Design) AND la.exact("English") AND pd(>20060711))) OR ((all(user-cent* design) AND la.exact("English") AND pd(>20060711)) OR (all(user-centered design) AND la.exact("English") AND pd(>20060711)) OR (all(user-centred design) AND la.exact("English") AND pd(>20060711)) OR (all(user-centred design process) AND la.exact("English") AND pd(>20060711)))) AND (all(Health* PRE/1 outcome*) AND la.exact("English") AND pd(>20060711))

**Limits:** la.exact("English") AND pd(>20060711)

**Dissertations and Theses (ProQuest)** on July 11, 2016

((all("Human-Cent* Design" OR "Human Centered Design" OR "Human Centred Design") OR (all(design/thinking) OR all(design NEAR/1 thinking)) OR all("User-Cent* Design" OR "User Centered Design" OR "User Centred Design")) AND la.exact("English") AND pd(> = 20060819)) AND (all(Health* AND outcome*) AND la.exact("English") AND pd(> = 20060819))

**Limits:** la.exact("English") AND after July 11, 2006

**Sociological Abstracts (ProQuest)** on July 12, 2016

(((design PRE/0 thinking) AND la.exact("English") AND pd(>20060711)) OR (((Human-Cent* Design) AND la.exact("English") AND pd(>20060711)) OR ((Human-Centered Design) AND la.exact("English") AND pd(>20060711)) OR ((Human-Centred Design) AND la.exact("English") AND pd(>20060711))) OR (((User-Centered Design) AND la.exact("English") AND pd(>20060711)) OR ((User-Centred Design) AND la.exact("English") AND pd(>20060711)) OR ((User-Cent* Design) AND la.exact("English") AND pd(>20060711)))) AND (health* AND la.exact("English") AND pd(>20060711))

**Limits**: la.exact("English") AND after July 11, 2006

**Social Service Abstracts (ProQuest)** on July 12, 2016

(("design thinking" AND la.exact("English") AND pd(>20060711)) OR (((Human Cent* PRE/1 Design) AND la.exact("English") AND pd(>20060711)) OR ((Human-Cent* PRE/1 Design) AND la.exact("English") AND pd(>20060711)) OR ((Human-Cent* Design) AND la.exact("English") AND pd(>20060711)) OR ((Human Cent* Design) AND la.exact("English") AND pd(>20060711)) OR ((Human-Centered Design) AND la.exact("English") AND pd(>20060711)) OR (Human Centered Design AND pd(>20060711)) OR ((Human-Centred Design) AND la.exact("English") AND pd(>20060711)) OR ((Human Centred Design) AND la.exact("English") AND pd(>20060711)) OR ((Human-Centred Design) AND la.exact("English") AND pd(>20060711))) OR ((User Cent* PRE/1 Design AND pd(>20060711)) OR (User-Cent* PRE/1 Design AND pd(>20060711)) OR ((User-Cent* Design) AND la.exact("English") AND pd(>20060711)) OR ((User Cent* Design) AND la.exact("English") AND pd(>20060711)) OR ((User-Centered Design) AND la.exact("English") AND pd(>20060711)) OR ((user centered design) AND la.exact("English") AND pd(>20060711)) OR ((user-centred design) AND stype.exact("Conference Papers & Proceedings") AND la.exact("English") AND pd(>20060711)) OR ((user centred design) AND la.exact("English") AND pd(>20060711)))) AND ((Health* PRE/1 outcome*) AND la.exact("English") AND pd(>20060711))

**Limits:** la.exact("English") AND after July 11, 2006

**Social Science(ProQuest)** on July 11, 2016

((((Design PRE/0 Thinking) AND la.exact("English") AND pd(>20060711)) OR ((Design PRE/1 Thinking) AND la.exact("English") AND pd(>20060711)) OR ((Design Thinking) AND la.exact("English") AND pd(>20060711))) OR (((Human Cent* PRE/1 Design) AND la.exact("English") AND pd(>20060711)) OR ((Human-Cent* PRE/1 Design) AND la.exact("English") AND pd(>20060711)) OR ((Human-Cent* Design) AND la.exact("English") AND pd(>20060711)) OR ((Human Cent* Design) AND la.exact("English") AND pd(>20060711)) OR ((Human-Centered Design) AND la.exact("English") AND pd(>20060711)) OR ((Human Centered Design) AND la.exact("English") AND pd(>20060711)) OR ((Human-Centred Design) AND la.exact("English") AND pd(>20060711)) OR ((Human Centred Design) AND la.exact("English") AND pd(>20060711))) OR (((User Cent* PRE/1 Design) AND la.exact("English") AND pd(>20060711)) OR ((User-Cent* PRE/1 Design) AND la.exact("English") AND pd(>20060711)) OR ((User-Cent* Design) AND la.exact("English") AND pd(>20060711)) OR ((User Cent* Design) AND la.exact("English") AND pd(>20060711)) OR ((User-Centered Design) AND la.exact("English") AND pd(>20060711)) OR ((user centered design) AND la.exact("English") AND pd(>20060711)) OR ((user-centred design) AND la.exact("English") AND pd(>20060711)) OR ((user centred design) AND la.exact("English") AND pd(>20060711)))) AND (Health* PRE/1 outcome*)

**Limits:** la.exact("English") AND after July 11, 2006

**SocINEX(EBSCOHost)** on July 11, 2016

S1. “Design w0 Thinking” OR Design w0 Thinking OR “Design w1 Thinking” OR Design w1 Thinking OR “Design Thinking” OR Design Thinking
S2. “Human Cent* W1 Design” OR Human Cent* W1 Design OR “Human-Cent* Design” OR “Human Cent* Design” OR “Human-Centered Design” OR “Human Centered Design” OR “Human-Centred Design” OR “Human Centred Design”
S3. “User Cent* w1 Design” OR “User-Cent* w1 Design” OR “User-Cent* Design” = OR “User Cent* Design” OR “User-Centered Design” OR “user centered design” OR “user-centred design” OR “user centred design” OR user centred design
S4. Health* W1 outcome*
S5. Outcome*
S6. s4 AND s5
S7. s1 OR s2 OR s3
S8. s6 AND s7

**Limits:**—Date of Publication: 20060701–20160731; Language: English; Search modes—Find all my search terms
